# Supplementary material for: Increased structural connectivity in high schizotypy
Source: Netw Neurosci. 2023 Jan 1;7(1):213–33. doi: 10.1162/netn_a_00279 (PMC10270715; doi:10.1162/netn_a_00279)
Supplement: Supplementary file 1 [file netn-7-1-213-s001.pdf]

Messaritaki, E., Foley, S., Barawi, K., Ettinger, U. & Jones, D. K. (2023).
Supporting information for “Increased structural connectivity in high schizotypy.”
Network Neuroscience, *7*(1), 213–233. https://doi.org/10.1162/netn_a_00279

Supporting Information

**Dataset / Atlas**	**Metric**	**Brain area**	**Correlation coefficient**	***p*-value**
**Cardiff data / AAL Atlas**	**Sensorimotor, mean node degree**	R Middle Frontal	0.18	0.037
		R Postcentral	0.18	0.045
	**Sensorimotor, mean clustering coefficient**	R Precentral	0.18	0.046
		R Postcentral	0.20	0.024
	**Default-mode, mean node degree**	L Middle Frontal	0.24	0.007
		R Inferior Frontal (Opercular)	***0.30***	***5×10^{-4}***
		L Inferior Frontal (Triangular)	0.21	0.018
		L Inferior Frontal (Orbital)	0.18	0.046
		L Middle Cingulate	***0.29***	***9×10^{-4}***
		R Middle Cingulate	0.22	0.011
		L Hippocampus	***0.29***	***7×10^{-4}***
		L Thalamus	0.18	0.041
	**Default-mode,**	L Superior Frontal	0.18	0.037

	**mean clustering coefficient**	***R Superior Frontal***	***0.24***	***0.006***
		L Inferior Frontal (Triangular)	***0.36***	***4 x 10^{-5}***
		L Anterior Cingulate	***0.29***	***8 x 10^{-4}***
		L Middle Cingulate	0.19	0.035
		R Middle Cingulate	***0.23***	***0.009***
		L Posterior Cingulate	0.19	0.033
		L Parahippocampal	0.18	0.048
		L Middle Occipital	0.18	0.046
		L Angular	***0.24***	***0.005***
		L Precuneus	***0.34***	***10^{-4}***
		R Precuneus	***0.40***	***4 x 10^{-6}***
**Cardiff data / Desikan-Killiany Atlas**	**Sensorimotor, mean node degree**	L Paracentral	0.22	0.012
		R Postcentral	0.21	0.015
		R Superior Frontal	0.21	0.017
	**Default-mode, mean node degree**	***L Caudal Middle Frontal***	***0.31***	***3 x 10^{-4}***
		L Isthmus Cingulate	***0.24***	***0.005***
		L Lateral Orbitofrontal	0.18	0.044

		L Parahippocampal	0.19	0.033
		L Precuneus	***0.30***	***5 x 10^{-4}***
		L Thalamus	0.20	0.021
		R Thalamus	0.20	0.020
		R Hippocampus	***0.26***	***0.003***
		R Parahippocampal	0.17	0.048
		R Pars Triangularis	***0.24***	***0.007***
		R Precuneus	***0.29***	***0.001***
		R Rostral Anterior Cingulate	0.20	0.019
**Munich data / Desikan-Killiany Atlas**	**Sensorimotor, mean node degree**	***L Paracentral***	***0.26***	***0.006***
		L Postcentral	***0.25***	***0.008***
		L Supramarginal	0.20	0.038
		R Postcentral	***0.27***	***0.006***
		R Precentral	***0.24***	***0.014***
		R Superior Frontal	0.20	0.039
	**Visual, mean node degree**	L Lingual	0.22	0.027
		R Cuneus	0.29	0.044
		R Fusiform	0.21	0.034

Table S1: Correlation coefficients and *p*-values between schizotypy score and the graph theoretical measure (node degree or clustering coefficient respectively) of the nodes that drive the correlations. (L = left, R = right). The correlation coefficients and *p*-values that survive multiple comparison correction are denoted in bold italic font.
